# Field‐based adipose tissue quantification in sea turtles using bioelectrical impedance spectroscopy validated with CT scans and deep learning

**DOI:** 10.1002/ece3.9610

**Published:** 2022-12-13

**Authors:** Sara Kophamel, Leigh C. Ward, Dmitry A. Konovalov, Diana Mendez, Ellen Ariel, Nathan Cassidy, Ian Bell, María T. Balastegui Martínez, Suzanne L. Munns

**Affiliations:** ^1^ College of Public Health, Medical and Veterinary Sciences James Cook University Townsville Queensland Australia; ^2^ School of Chemistry and Molecular Biosciences The University of Queensland St Lucia Queensland Australia; ^3^ College of Science and Engineering James Cook University Townsville Queensland Australia; ^4^ Australian Institute of Tropical Health and Medicine Townsville Queensland Australia; ^5^ North Queensland X‐Ray Services Townsville Queensland Australia; ^6^ Department of Environment and Science Queensland Government Townsville Queensland Australia; ^7^ Department of Animal Medicine and Surgery CEU Cardenal Herrera University, CEU Universities Valencia Spain

**Keywords:** adipose tissue, Bland–Altman, body condition, body fat, nutritional status, sea turtle

## Abstract

Loss of adipose tissue in vertebrate wildlife species is indicative of decreased nutritional and health status and is linked to environmental stress and diseases. Body condition indices (BCI) are commonly used in ecological studies to estimate adipose tissue mass across wildlife populations. However, these indices have poor predictive power, which poses the need for quantitative methods for improved population assessments. Here, we calibrate bioelectrical impedance spectroscopy (BIS) as an alternative approach for assessing the nutritional status of vertebrate wildlife in ecological studies. BIS is a portable technology that can estimate body composition from measurements of body impedance and is widely used in humans. BIS is a predictive technique that requires calibration using a reference body composition method. Using sea turtles as model organisms, we propose a calibration protocol using computed tomography (CT) scans, with the prediction equation being: adipose tissue mass (kg) = body mass − (−0.03 [intercept] − 0.29 * length^2^/resistance at 50 kHz + 1.07 * body mass − 0.11 * time after capture). CT imaging allows for the quantification of body fat. However, processing the images manually is prohibitive due to the extensive time requirement. Using a form of artificial intelligence (AI), we trained a computer model to identify and quantify nonadipose tissue from the CT images, and adipose tissue was determined by the difference in body mass. This process enabled estimating adipose tissue mass from bioelectrical impedance measurements. The predictive performance of the model was built on 2/3 samples and tested against 1/3 samples. Prediction of adipose tissue percentage had greater accuracy when including impedance parameters (mean bias = 0.11%–0.61%) as predictor variables, compared with using body mass alone (mean bias = 6.35%). Our standardized BIS protocol improves on conventional body composition assessment methods (e.g., BCI) by quantifying adipose tissue mass. The protocol can be applied to other species for the validation of BIS and to provide robust information on the nutritional and health status of wildlife, which, in turn, can be used to inform conservation decisions at the management level.

## Graphical abstract

Loss of body fat (i.e., adipose tissue) in vertebrate wildlife species is indicative of decreased nutritional and health status and is linked to environmental stress and diseases. Here, we calibrate bioelectrical impedance spectroscopy (BIS) for assessing the nutritional status of sea turtles. Using a form of artificial intelligence, we trained a computer model to identify and quantify body fat from computed tomography scans, which enabled estimating body fat from the BIS measurements. Our standardized BIS protocol can be applied to other species and taxa and improves on conventional body composition assessment methods (e.g., body condition indices) by accurately estimating body fat.

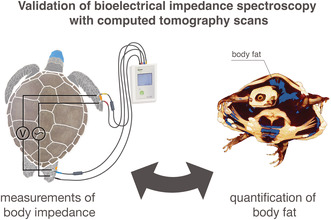

## 1 INTRODUCTION

### 1.1 Conventional methods for body composition assessment

Assessing body composition is an integral component of ecological, behavioral, and evolutionary studies in vertebrate wildlife. The macro‐composition of the body (i.e., adipose tissue and nonadipose tissue) changes when nutritional intake is mismatched with the nutritional requirements (Ward, 2018a). Fat, primarily adipose tissue, is the primary energy store in vertebrates. The mobilization of energy reserves is especially important in vertebrate animals exposed to prolonged fasting, or in females undergoing vitellogenesis (Hamann et al., 2002; Lignot & LeMaho, 2012). The loss of adipose tissue has also been linked to chronic stressors such as anthropogenic and environmental threats, climate change, and diseases (Karasov & del Rio, 2020; Price & Valencak, 2012). The standard, field‐based method for assessing body composition in sea turtles is to determine body condition indices (BCI), such as Fulton's condition factor (*K* = body mass/straight carapace length^3^ * 10,000; Bjorndal et al., 2000; Harris et al., 2017). These indices are simple to obtain and do not require extensive training (Harris et al., 2017; Wilder et al., 2016) but have poor predictive power for quantifying adipose tissue (Stevenson & Woods Jr., 2006; Wilder et al., 2016). Robust alternatives that can be applied in the field would enhance our understanding of sea turtle population health.

### 1.2 Bioelectrical impedance spectroscopy

Bioelectrical impedance spectroscopy (BIS), which falls under the category of bioelectrical impedance analysis (BIA) methods, is considered an accurate, portable, quick, affordable, and noninvasive method that has been used to predict body composition in humans (Lemos & Gallagher, 2017; Ward, 2018b), fishes (Ćurić et al., 2017), and in domestic and laboratory animal research (Muller et al., 2021; Ward et al., 2009). BIS measures the opposition of biological tissues to the flow of an electric current (impedance). The resulting impedance values (or more correctly its component, resistance) are used, in combination with body mass and length measurements, to predict nonadipose tissue. Adipose tissue is then calculated from the nonadipose tissue estimates by difference with body mass (Van Marken Lichtenbelt, 2001). For additional information on the rationale behind the chosen impedance parameters and on how to conduct impedance measurements on sea turtles, we refer the interested reader to Kophamel et al. (2023). The portability and ease of use of BIS devices make them especially attractive for assessing threatened species in the field. However, the successful application of BIS for wildlife assessments requires the adoption of standardized protocols, identification and control for potential confounding factors, and appropriate calibration and validation (Haus et al., 2017; Kophamel et al., 2023; Ward et al., 2009). Noninvasive methods for body composition assessment, such as diagnostic imaging tools, were found to strongly correlate with the reference calibration method (i.e., chemical analyses). Diagnostic imaging tools are therefore suitable for BIS calibration where chemical analyses of threatened species are undesirable (Ross et al., 1991; Wyatt et al., 2015).

Integrating adipose tissue data in sea turtle monitoring programs could help to identify drivers of population declines or measure the effectiveness of a conservation program (IUCN – SSC Species Conservation Planning Sub‐Committee, 2017). Nutritional status assessment can serve as indicators for population decrease and population viability (Deem et al., 2001; Page‐Karjian et al., 2020). Monitoring nutritional status, in combination with foraging ground assessments, population abundance, and demographic parameters, can provide an early warning about potential anthropogenic and environmental threats, which might have long‐lasting consequences on health status and on the turtles' habitat (Deem & Harris, 2017). The combined and simultaneous monitoring of both sea turtle habitat and nutritional status might thus provide a clearer picture of the impacts of threats on foraging and nesting sites, and on sea turtle population health. The integration of monitoring programs with adipose tissue data may also serve as a guidance for state agencies, researchers, and NGOs wishing to enhance conservation efforts on other threatened species.

The aims of this study were to conduct paired BIS and computed tomography (CT) measurements on a model species (green turtle, *Chelonia mydas*), to develop a fully automated process for adipose tissue identification and quantification from the CT scans by using a form of artificial intelligence (i.e., convolutional neural networks, CNN), and to use these data to calibrate a BIS body composition device for field‐use. This study also provides a novel automated protocol for CT image processing that can be adapted to other sea turtle species and to other taxa.

## 2 MATERIALS AND METHODS

### 2.1 Animals

This study was conducted in North Queensland, Australia, between June 2019 and March 2021. The sample consisted of *n =* 49 green turtles (*Chelonia mydas*): *n =* 25 wild and immature turtles caught from Cleveland Bay (19°13′05″S, 146°55′19″E) and Toolakea Beach (19°08′40″S, 146°34′40″E), and *n =* 24 captive turtles sourced from the Turtle Health Research Facility at James Cook University that had been transferred from Heron Island (Queensland, Australia). See Kophamel et al. (2023) for husbandry details. Turtles were transported to shore immediately after capture. The sex of the captured turtles was unknown. The inclusion criteria for all animals were to be clinically healthy and to have a carapace width of less than 55 cm (width limitation of the CT scanner gantry). A complete physical examination was performed by a qualified veterinarian (SK) and only animals that appeared healthy, in a good body condition (visual assessment), and without evident injuries, tumors, or limb amputations were used in the study. Impedance devices assume a constant hydration fraction and a certain body geometry (i.e., individuals with four limbs present; Kophamel et al., 2023). Lethargic animals may not be normally hydrated, and turtles with missing limbs will have a different body geometry than turtles with all limbs present. These characteristics could alter the impedance measurements, which underscores the importance of excluding dehydrated animals and animals with missing limbs when performing a calibration study unless these limitations are accounted for in the predictive models.

The health status of each turtle was further examined with biochemical and hematological analyses, which are detailed in Kophamel, Rudd et al. (2022). The curved carapace length (CCL) and straight carapace length (SCL) were measured twice from the nuchal scute to the caudal tip of the supracaudal scute, to the nearest millimeter, with the average value being recorded. Body mass was measured to the nearest 0.01 kilogram (kg) by suspending each turtle in a custom harness from a digital hanging scale. Body temperature was measured using a thermocouple (8402‐20 Thermistor 237 Thermometer, Cole‐Palmer Instruments), and by inserting the probe 5 cm into the cloaca (Flint, 2013; Stacy & Innis, 2017). Animal characteristics are detailed in the Appendix S1, Table A2.

All experimental procedures were completed within the same day and were approved by Animal Ethics (permit number A2525), the Great Barrier Reef Marine Park Authority (permit numbers G18/40749.1 and G19/42769.1), and the Department of Environment and Science, Queensland Government (permit numbers SPP18‐001167 and SPP18‐001167‐1).

### 2.2 Bioelectrical impedance spectroscopy

**FIGURE 1:**
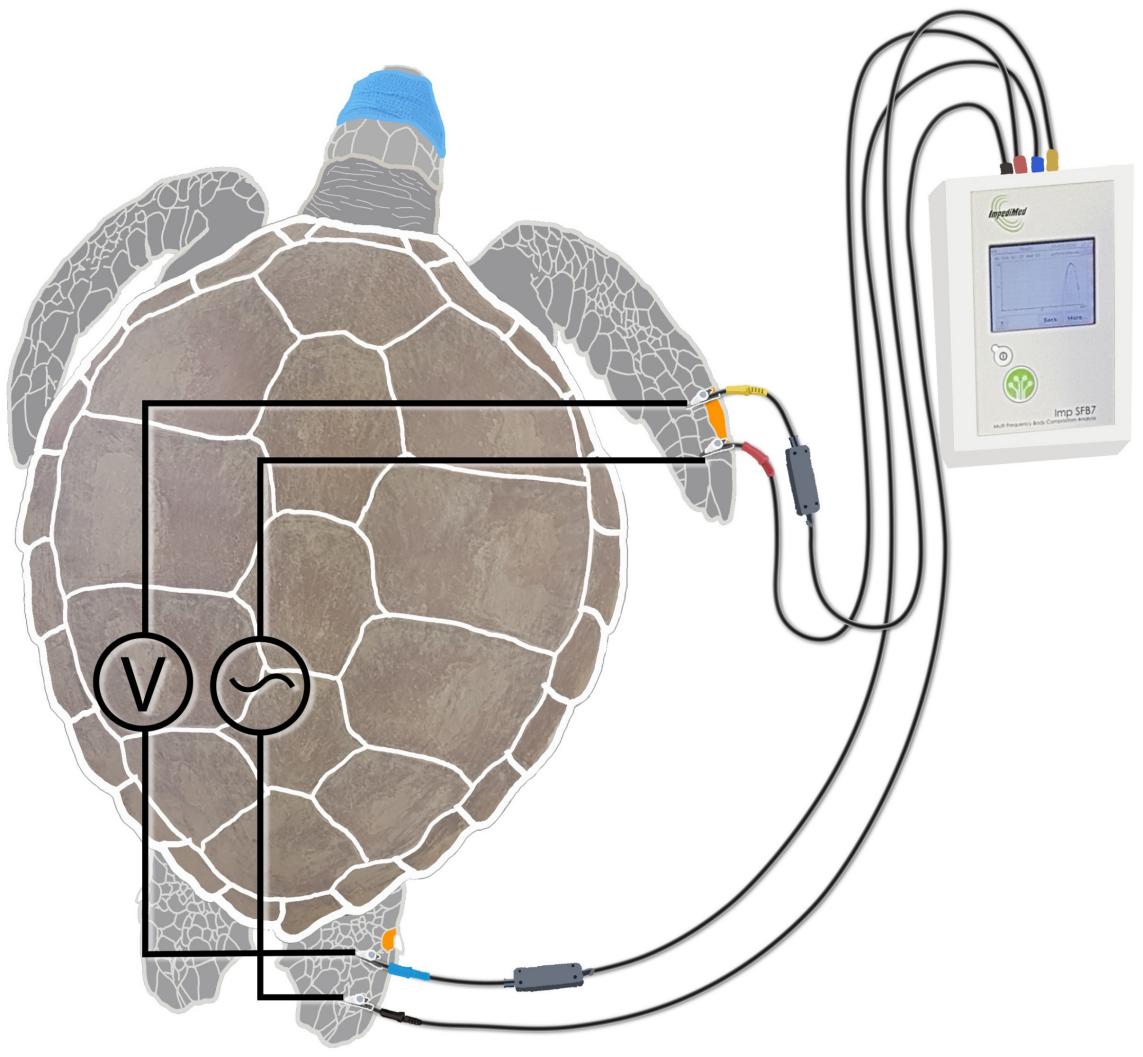
Anatomical locations for placing bioelectrical impedance spectroscopy (BIS) electrode needles on a juvenile green turtle (*Chelonia mydas*), using a handheld SFB7 BIS device. Electrode placement was standardized at consistent anatomic markers. Distally placed electrodes (red and black) introduce the current and proximally placed electrodes (yellow and blue) record voltage using a high input impedance voltmeter. Current‐receiving and current‐introducing electrodes are inserted 2 mm subdermally and placed ≥3 cm apart to avoid current inferences. Anatomical positions of the electrodes are standardized using reference scales (orange). On the right front limb, the longest scale at the limb periphery is used as a reference. The recording electrode (yellow) is placed at the medial side of this scale and the current‐introducing electrode (red) is placed at the distal margin. In the right hind limb, the scale medial to the claw is used as a reference. The recording electrode (blue) is placed at the medial side of this scale, and the current‐introducing electrode (black) is placed at the distal margin. Figure sourced from Kophamel et al. (2023).

Bioelectrical impedance measurements were performed using a BIS device (SFB7, Impedimed), that measures resistance and reactance to an applied harmless, alternating electric current at 256 logarithmically‐spaced frequencies in the range of 3–1000 kHz. Device calibration was verified daily. BIS measurements were carried out 1.5 ± 2.0 h postcapture. Each animal was first placed prone on a nonconductive surface and their eyes covered with a nonconductive cohesive bandage to reduce stress. After disinfecting the skin with 70% ethanol, resistance measurements were taken by attaching electrode leads to two needles (27‐gauge × ½ inch needle, Terumo, Japan) inserted 2 mm sub‐dermally in the right forelimb and in the right hindlimb (Figure 1), following the methods described in Kophamel et al. (2023). Electrodes were 3 cm apart, with the distal electrode applying the current and the proximal electrode sensing the voltage. Ten sequential measurements, with an interval of 5 s, were taken without removing the electrodes. The complete procedure, from animal preparation and examination to impedance measurements, took no longer than 15 min per animal and did not require anesthetizing or sedating the animals. See Kophamel et al. (2023) for full details on the BIS standardization procedure and precision (i.e., intra‐animal variability) estimates in sea turtles. The extracted data of interest were resistance at infinite frequency (Rinf, predictor of total body water and nonadipose tissue); resistance at zero frequency (R0, predictor of extracellular water); intracellular resistance (Ri, an index of intracellular water); and, for comparison with studies using the more affordable single‐frequency (50 kHz) impedance devices, resistance at 50 kHz (R50), reactance at 50 kHz (Xc50), and phase angle at 50 kHz (PhA50). Resistance data are required to estimate nonadipose tissue, from which adipose tissue can be derived by difference in body mass.

### 2.3 Computed tomography scans

**FIGURE 2:**
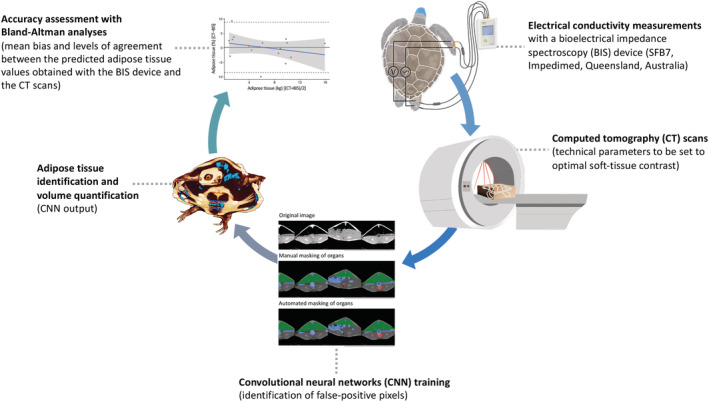
Standardization protocol for calibrating a bioelectrical impedance spectroscopy (BIS) device for adipose tissue quantification in green turtles (*Chelonia mydas*, *n =* 49) using whole‐body computed tomography (CT) scans. The calibration consists of five steps, which comprise (1) conducting electrical conductivity (i.e., body impedance) measurements with a BIS device; (2) performing CT scans on the animals that were assessed with the BIS device; (3) training a convolutional neural networks (CNN) model on the CT scans to automate the identification of false‐positive pixels; (4) identifying and quantifying the adipose tissue volume from the CNN model; and (5) assessing the accuracy of the calibration with Bland–Altman analyses. For additional information on the technical details behind bioelectrical impedance measurements in sea turtles, we refer the interested reader to Kophamel et al. (2023).

Each turtle was secured in the prone position by wrapping loosely in a towel and placing within a cardboard box (Figure 2). Eyes were covered with a cohesive bandage to reduce visual stimuli and stress. Optimal soft‐tissue contrast was achieved using a peak kilovoltage (kVp) of 120 and a tube current of 320 milliamperage (mA). Volumetric data of total body scans were acquired in helical scan mode, with 1.25 mm slice thickness and spacing between slices set at 0.625 mm (Optima CT660 16 slice scanner, GE Medical Systems; and Aquilion Lightning 160). The typical number of CT slices per animal was around 1600. In this study, the total number of slices was approximately 80,000, and approximately 50,000 CT slices were used in the final calculations. Turtles were released 4 to 5 h postcapture; wild turtles were returned to their capture location, and captive turtles returned to their usual housing.

### 2.4 Automated adipose tissue quantification

Adipose tissue Hounsfield units (HU; i.e., attenuation ranges) were identified by three‐dimensional rendering using a commercially available, validated software for DICOM (Digital Imaging and Communications in Medicine) visualization and body composition analysis (NovaPACS, Novarad™; Appendix S1, Figure A1), following the methods described in Gibby et al. (2017), Depersio et al. (2019), and Newman et al. (2019). The location of adipose tissue on the CT scans was confirmed with the previous necropsy information from stranded and euthanized animals and with the identification criteria provided by other authors (Stacy et al., 2017; Valente, 2008; Wyneken, 2001). A two‐step hybrid approach was then implemented to perform the fully automated adipose tissue quantification.

In the first step, the direct per‐pixel HU‐thresholding yielded segmentation masks with negligible areas of false negatives (i.e., adipose tissue areas identified as nonadipose tissue), and with significant areas of false positives (i.e., nonadipose tissue areas identified as adipose tissue). False positives were further divided into four groups (referred to as ABCD masks from now on): (A) skin folds, eyes, the epidural space, and artifacts resulting from metal identification tags; (B) transition from the respiratory tract to pulmonary soft‐tissue areas; (C) pericardial region (i.e., heart region); and (D) gastrointestinal tract (GIT) contents. The CT scanner table was also falsely identified as adipose tissue.

**FIGURE 3:**
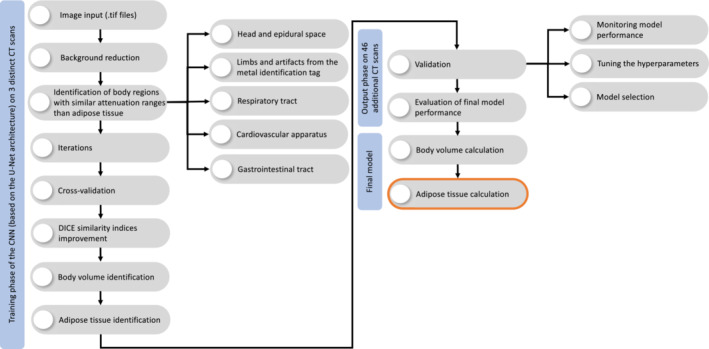
Flowchart and workflow to create an automated adipose tissue quantification in green turtles (*Chelonia mydas*, *n =* 49) using whole‐body computed tomography (CT) scans and convolutional neural networks (CNN). The workflow consists of (1) training the CNN model for adipose tissue identification from the flowchart and workflow to create an automated adipose tissue quantification in green turtles (*Chelonia mydas*, *n =* 49) using whole‐body CT scans and CNN. The workflow consists of (1) training the CNN model for adipose tissue identification from the CT scans; (2) validating and evaluating the model performance; and (3) building the final model, which enables the calculation of total adipose tissue volume, from which adipose tissue mass can be derived. Please refer to Zopfs et al. (2020) for a detailed explanation of the generic CNN model for adipose tissue identification and quantification.

In the second step, the deep learning semantic‐segmentation CNNs were trained to identify the false‐positive pixels (i.e., nonadipose tissue) using the widely accepted U‐Net semantic‐segmentation CNN architecture (Figure 3; Ronneberger et al., 2015). An open‐sourced machine learning framework (PyTorch) was used for implementing the U‐Net (Paszke et al., 2019) and was adapted from Yakubovskiy (2020). U‐Nets were trained by segmenting three training masks for each of the three animals representing small (34.4 cm SCL), medium (46.5 cm SCL), and large size (56.4 cm SCL) animals. For each of the three animals, every second CT slice was enlarged two‐fold, from the original 512 × 512 pixels to a 1024 × 1024 gray‐scale image, and manually segmented into ABCD masks (Figures 2 and 3). As the table‐related pixels were very similar between slices, every 20th slice was segmented for identifying the false‐positive pixels from the CT scanner table. Segmentation resulted in a total of 6292 ABCD masks and 151 table masks. U‐Nets were trained for the CT scanner table masks, the GIT masks (D mask), and the combined ABC masks. Due to the simple geometrical shape of the CT table, an 11‐layer VGG‐based (Visual Geometry Group) CNN classification was used as an image features encoder in the CT table U‐Net (vgg‐11bn PyTorch ImageNet pretrained version; Simonyan & Zisserman, 2014). By contrast, a 34‐layer ResNet CNN was used as the image features encoder in the D mask and ABC masks U‐Nets (He et al., 2016). Due to the required high segmentation accuracy, each final network version consumed approximately 1 week of continuous training on a single NVIDIA GTX1080 GPU available for this project. Technical details of the training are beyond the scope of this publication and will be published separately. Total body volume and adipose tissue volume (in cm^3^ and as %) were determined and adipose tissue mass (kg) calculated by multiplying the total body mass (as measured by digital scale) by the fractional adipose tissue percentage.

### 2.5 Statistical analyses

Data are presented as mean ± SD or median with interquartile range, as appropriate. The HU ranges for adipose tissue quantification by CT scans were determined using generalized additive models. Model selection and validation for HU ranges were conducted as described below. Stepwise multiple linear regression analysis was used to develop the prediction equations for nonadipose tissue mass estimations, from which adipose tissue mass was derived by difference with body mass. The dependent variable was adipose tissue mass (% and kg) as predicted by the automated CT scan method (i.e., criterion method), and the predictor variables were impedance indices calculated with SCL and CCL as measures for body length (i.e., length^2^/Rinf, length^2^/R0, length^2^/R50, length^2^/Ri, and length^2^/Xc50), total body mass (kg), and time after capture (h) (Kophamel et al., 2023). Tukey's post‐hoc multiple comparison tests were conducted to assess the effects of predictor variables on the dependent variable (R package emmeans, α = 0.05; Lenth, 2016). The final model selections were based on the corrected Akaike Information Criterion (Barton & Barton, 2015), on diagnostic residual plots, and on the fit of the data to the selected model. Predictive power of the selected model was examined by refitting the model on a randomized subsample (1/3 of the original sample size). Additional correlations between variables were assessed using the concordance correlation coefficient and Pearson's correlation coefficient (strong correlation assumed when *p* < .05 and *r* > .5).

Equation accuracy was assessed using Bland–Altman analyses with 95% levels of agreement (LOA), which display mean bias and LOA between the predicted adipose tissue values obtained with the CT and the BIS methods (Table 1; Appendix S1, Figure A2). The Bland and Altman plot assesses whether the mean adipose tissue value for a population (estimated with the BIS method) is close to the measured reference adipose tissue value (CT method). More specifically, the plot quantifies the bias (i.e., predictive error) and range (i.e., limits) of agreement that includes 95% of the differences between two methods (Altman & Bland, 1983; Bland & Altman, 1986). Mean bias refers to the accuracy of adipose tissue mass predictions at the population level, and lower and upper LOA refer to the individual level (i.e., an individual could be anywhere within the 95% LOA limits). Smaller mean bias and narrower LOA values imply higher accuracy and a lower magnitude of error for adipose tissue estimation by the BIS method at the population and individual levels. If the mean bias is narrow, but the LOA limits are wide, accuracy at the population level will still be high, but since the margin of error in the predicted adipose tissue value for an individual is wide, the accuracy of the adipose tissue mass prediction at the individual level will be reduced. Precision (i.e., intra‐animal variability) estimates of the impedance measurements, which determine whether BIS is suitable for estimating adipose tissue changes in individual turtles over time, are detailed in Kophamel et al. (2023).

**TABLE 1:** Equation coefficients used to estimate nonadipose tissue mass (kg) in green turtles (*Chelonia mydas*; *n =* 49), 95% limits of agreement for adipose tissue mass prediction, maximum allowed difference, concordance correlation coefficient (*r*_c_), Pearson's correlation coefficient (*r*_p_), and median absolute percentage error (MAPE) results.

Parameter	Coefficients for nonadipose tissue mass (kg) prediction (*n =* 33/49); corresponding values for total population (*n =* 49) in parentheses				*R* ^2^	RMSE (df)	F statistic (df)	95% limits of agreement for adipose tissue mass^a^ (absolute values in kg (%))					*r*_c_	*r*_p_	MAPE (median)
	Intercept	Impedance Index (length^2^/R)	Body mass (kg)	Time after capture (h)				Mean bias^b^	SD of the mean bias	Lower^b^	Upper^b^	Max. allowed difference^c^			
SCL^2^/Rinf	−0.02 *(0.01)*	−0.11 *(−0.10)*	1.01 *(1.00)*	−0.13 *(−0.10)*	0.998 *(0.998)*	0.24 (29)	4495 (3, 29)	0.06 (0.18%)	0.25 (0.79%)	−0.42 (−8.59%)	0.54 (9.13%)	0.73	0.998	0.998	3.28
CCL^2^/Rinf	−0.01 *(0.02)*	−0.11 *(−0.14)*	1.01 *(1.02)*	−0.13 *(−0.10)*	0.997 *(0.997)*	0.24 (29)	4475 (3, 29)	0.06 (0.28%)	0.25 (1.25%)	−0.43 (−8.73%)	0.54 (9.46%)	0.73	0.998	0.998	3.38
SCL^2^/R0	−0.04 *(−0.02)*	−0.44 *(−0.41)*	1.12 *(1.10)*	−0.12 *(−0.08)*	0.998 *(0.997)*	0.19 (29)	6557 (3, 29)	0.03 (0.47%)	0.25 (3.71%)	−0.45 (−8.71%)	0.52 (9.89%)	0.71	0.998	0.998	2.40
CCL^2^/R0	−0.03 *(−0.01)*	−0.43 *(−0.39)*	1.12 *(1.10)*	−0.12 *(−0.08)*	0.998 *(0.997)*	0.20 (29)	6485 (3, 29)	0.03 (0.61%)	0.25 (5.02%)	−0.46 (−8.82%)	0.52 (10.24%)	0.71	0.998	0.998	3.59
**SCL** ^**2**^ **/R50**	**−0.06 *(−0.03)***	**−0.32 *(−0.29)***	**1.09 *(1.07)***	**−0.14 *(−0.11)***	**0.999 *(0.998)***	**0.19 (29)**	**6927 (3, 29)**	**0.04 (0.11%)**	**0.25 (0.76%)**	**−0.46 (−8.52%)**	**0.53 (8.95%)**	**0.72**	**0.998**	**0.998**	**3.23**
**CCL** ^**2**^ **/R50**	**−0.04 *(−0.01)***	**−0.30 *(−0.28)***	**1.09 *(1.07)***	**−0.14 *(−0.10)***	**0.998 *(0.998)***	**0.19 (29)**	**6792 (3, 29)**	**0.03 (0.22%)**	**0.25 (1.73%)**	**−0.46 (−9.06%)**	**0.53 (9.72%)**	**0.71**	**0.998**	**0.998**	**3.42**
SCL^2^/Ri	−0.00 *(0.00)*	−0.15 *(−0.37)*	0.98 *(1.01)*	−0.13 *(−0.13)*	0.997 *(0.997)*	0.27 (29)	3356 (3, 29)	0.05 (0.50%)	0.25 (2.38%)	−0.43 (−8.75%)	0.53 (9.86%)	0.72	0.998	0.998	3.66
CCL^2^/Ri	−0.01 *(0.01)*	−0.14 *(−0.36)*	0.98 *(1.01)*	−0.13 *(−0.13)*	0.997 *(0.997)*	0.27 (29)	3372 (3, 29)	0.05 (0.48%)	0.25 (2.33%)	−0.43 (−8.80%)	0.54 (9.97%)	0.72	0.998	0.998	3.65
Body mass	−0.06 *(0.11)*	NA *(NA)*	0.97 *(0.92)*	NA (*NA*)	0.996 *(0.994)*	0.02 (13)	3144 (1, 13)	−0.21 (6.35%)	0.36 (11.43%)	−0.92 (−4.93%)	0.50 (9.40%)	1.18	0.991	0.998	23.59

*Note*: Impedance index (length^2^/resistance R) was calculated using straight and curved carapace length (SCL and CCL), resistance at infinite frequency (Rinf), resistance at zero frequency (R0), resistance at 50 kHz (R50), intracellular resistance (Ri), and reactance at 50 kHz (Xc50). Most accurate coefficients (i.e., smallest mean bias and lower and upper 95% limits of agreement) are highlighted in bold. Please refer to the Appendix S1, Figure A2 for Bland and Altman plots using the coefficients described below.

Abbreviations: CCL, curved carapace length; SCL, straight carapace length.

^a^ Calculated as: Total body mass (weight in kg) ‐ nonadipose tissue mass (kg).

^b^ Mean bias refers to adipose tissue mass predictions at the population level. Lower and upper 95% limits of agreement refer to adipose tissue mass predictions at the individual level.

^c^ The maximum allowed difference indicates the predefined agreement level for a sample size of *n =* 49 at 80% power and α = 0.05. Differences below this limit are irrelevant or neglectable (Lu et al., 2016). Two methods are considered to be in agreement when a predefined maximum allowed difference (Δ) is larger than the higher limit of agreement, and ‐Δ is lower than the lower limit of agreement.

The accuracy of adipose tissue predictions by the BIS method was further compared using mean absolute percentage error (MAPE; Table 1), Passing and Bablok regressions (Appendix S1, Figure A3), and maximum allowed difference, which indicated the predefined agreement level for a sample size of *n =* 49 at 80% power and α = 0.05 (Table 1). Differences below this limit are irrelevant or neglectable (Lu et al., 2016). The last step consisted in extrapolating the most accurate predictive model to the whole sample (*n =* 49/49), which resulted in a final prediction equation recommended for future studies. To assess the accuracy of body mass as sole predictor variable for adipose tissue mass estimation, an additional model using body mass as predictor variable (excluding impedance index) was created for comparison purposes with the best‐fitting model.

All statistical analyses were produced with R statistical software, using the package ggplot2 for data visualization (Hadley, 2016; R Core Team, 2019). The datasets for assessing the validity of our work (.xlsx, .ods., and .csv formats) are available at James Cook University Data Repository under the following link: https://doi.org/10.25903/gzf1‐8e56 [doi:10.25903/gzf1‐8e56] (Kophamel, Ward, et al., 2022). Description of the parameters used in the statistical models and a list of abbreviations are detailed in the appendices (Appendix S1, Figure A2, and Appendix S1, section “Description of parameters and codes used in the dataset”).

## 3 RESULTS

### 3.1 Adipose tissue attenuation ranges

The neck, sub‐carapace, mesenteric, and hindlimb regions were visually identified as the main contributors to adipose tissue mass. Individual adipose tissue HU ranged from mean HUmin = −32.2 ± 33.8 to mean HUmax = 10.1 ± 16.5 (*n =* 49), and mean adipose tissue resulting from the impedance measurements was estimated to be 6.5 ± 3.7%. Additional animal characteristics are displayed in the Appendix S1, Table A2.

### 3.2 Adipose tissue quantification

The models generated to predict adipose tissue mass from nonadipose tissue mass had an improved fit when impedance index (i.e., length^2^/resistance), morphometric data (i.e., body mass), and time after capture were included as explanatory variables (Table 1), as these variables have been shown to alter impedance measurements in green turtles, if not accounted for (Kophamel et al., 2023). The most accurate equation that resulted from the prediction group (*n =* 33/49) used the impedance index SCL^2^/R50 and had a mean bias of 0.11% and LOA of −8.52% to 8.95% (Equation 1, Figure 3; Table 1).

**The best‐fit equations for predicting adipose tissue mass from BIS measurements of impedance indices (a) SCL** ^**2**^ **/R50 and (b) CCL** ^**2**^ **/R50 in the prediction group (*n =* 33/49) of green turtles (*Chelonia mydas*).** SCL^2^/R50 and CCL^2^/R50 are the impedance index (length^2^/resistance at 50 kHz) calculated using SCL and CCL; body mass is the total weight (kg); and time after capture refers to the hours that have passed since capturing the animal.

$$\left(a\right)\;\text{Adipose tissue mass}\;\left(\mathrm{kg}\right)=\text{Body mass}-\left(-0.06\;\left[\text{intercept}\right]-0.32*{\mathrm{SCL}}^{2}/R50+1.09*\text{body mass}-0.14*\text{time after capture}\right)$$

(1) $$\left(b\right)\;\text{Adipose tissue mass}\;\left(\mathrm{kg}\right)=\text{Body mass}-\left(-0.04\;\left[\text{intercept}\right]-0.30*{\mathrm{CCL}}^{2}/R50+1.09*\text{body mass}-0.14*\text{time after capture}\right)$$

The impedance indices that resulted in the lowest error ranges were length^2^/resistance at infinite frequency (length^2^/Rinf), length^2^/resistance at 50 kHz (length^2^/R50), and length^2^/resistance at zero frequency (length^2^/R0), which are reflective of extracellular water (Rinf) and total body water (R0, R50) (Table 1). See Kophamel et al. (2023) for details on how to measure and interpret resistance parameters in sea turtles. The mean bias between CT and BIS estimations of adipose tissue ranged from 0.11% to 0.61%, and LOA ranged from −9.06% to 10.24% (predictor variables length^2^/Rinf, length^2^/R50, length^2^/R0, and length^2^/Ri; Table 1 and Appendix S1, Figure A2).

The BIS‐ and CT‐derived adipose tissue mass estimates were highly correlated (*R* ^2^ > 0.99), irrespective of the tested resistance parameters (Rinf, R50, R0, Ri). Summary statistics of the equation coefficients used to estimate adipose tissue mass with impedance indices length^2^/Rinf, length^2^/R50, length^2^/R0, and length^2^/Ri, and including CCL or SCL, are included in Table 1. The final prediction equation (Equation 2), which is recommended for use in future studies on green turtles, was generated by applying the best‐fit model to the whole sample (*n =* 49/49). By contrast, adipose tissue estimated from body mass alone resulted in a larger mean bias (6.35%, measure of population‐level accuracy) and wider LOA (−4.93% to 9.40%, measure of predictive accuracy in individual animals). Furthermore, the BIS method was over 50 times more accurate in predicting adipose tissue at the population level, compared with solely using body mass (mean biases of 0.11% and 6.35%, respectively).

**Final equations for predicting adipose tissue mass from BIS measurements of impedance indices (a) SCL** ^**2**^ **/R50 and (b) CCL** ^**2**^ **/R50 in green turtles (*Chelonia mydas*).** Equations (a) and (b) resulted from applying the most accurate prediction model (Table 1) to the whole sample (*n* = 49/49). SCL^2^/R50 and CCL^2^/R50 are the impedance index (length^2^/resistance at 50 kHz) calculated using SCL or CCL; body mass is the total weight (kg); and time after capture refers to the hours that have passed since capturing the animal.

$$\left(a\right)\;\text{Adipose tissue mass}\;\left(\mathrm{kg}\right)=\text{Body mass}-\left(-0.03\;\left[\text{intercept}\right]-0.29*{\mathrm{SCL}}^{2}/R50+1.07*\text{body mass}-0.11*\text{time after capture}\right)$$

(2) $$\left(b\right)\;\text{Adipose tissue mass}\;\left(\mathrm{kg}\right)=\text{Body mass}-\left(-0.01\;\left[\text{intercept}\right]-0.28*{\mathrm{CCL}}^{2}/R50+1.07*\text{body mass}-0.10*\text{time after capture}\right)$$

## 4 DISCUSSION

Bioelectrical impedance spectroscopy analysis enabled quantifying, analyzing, and interpreting body composition data in green turtles. We propose an automated in vivo quantification of adipose tissue mass using whole‐body CT scans. The use of CNN in a deep learning approach facilitated a fully automated body composition assessment. In addition, our suggested protocol can be used for standardizing adipose tissue quantification in other species and taxa.

**FIGURE 4:**
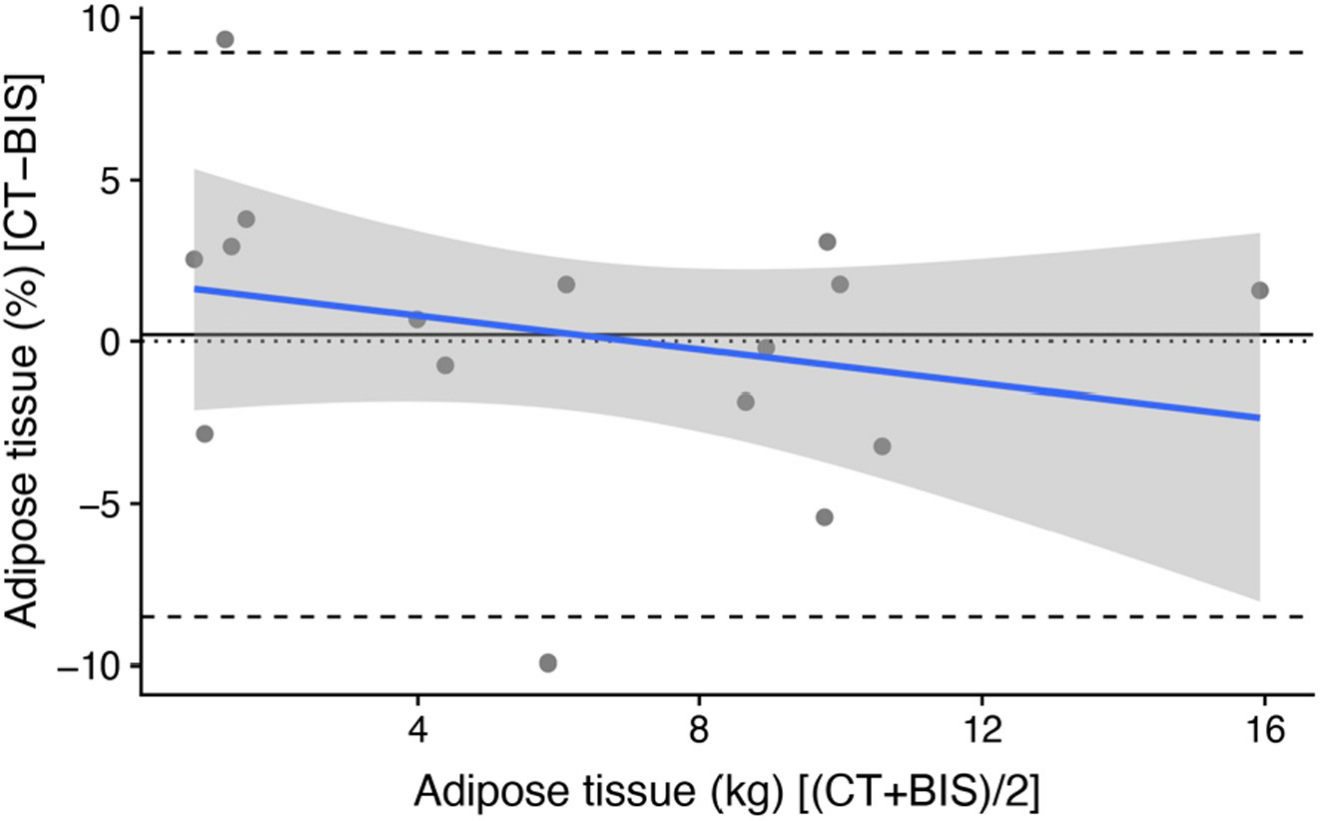
Bland and Altman plot (Altman & Bland, 1983; Bland & Altman, 1986) of the differences between adipose tissue (AT) estimates (%) from CT and bioelectrical impedance spectroscopy (BIS via SCL^2^/R50) (Y axis) and the mean of each pair of AT estimates (kg) from CT and BIS via SCL^2^/R50 (X axis) in green turtles (*Chelonia mydas*). There was a bias of 0.11 units (%) between the two AT estimates, which is the gap between the mean of the differences in AT estimates (black solid line) and the zero line (black dotted line), representing no mean difference in AT estimates. The 95% limits of agreement (LOA, black dashed lines) represent a 95% prediction interval, such that 95% of differences in AT estimated by the two methods fell between −8.52% and 8.95%. While the mean difference between methods (bias) represents accuracy at the population level, the limits of agreement indicate confidence in method agreement for an individual. The regression line (blue solid line) and its 95% confidence interval (gray area) show the relationship between the dependent variables on the Y axis (differences between AT estimates from CT and BIS measurements) and the independent variables on the X axis (mean of each pair of AT estimates from CT and BIS measurements). The lowest mean bias and LOA were generated using straight carapace length^2^/resistance at 50 kHz (SCL^2^/R50) as predictor variable (Table 1). Please refer to the Appendix S1, Figure A2 for Bland and Altman plots using other impedance parameters, and to Giavarina (2015) for a detailed explanation on how to use and interpret Bland and Altman plots.

Prediction of adipose tissue mass at the population level was highly accurate (mean bias of 0.11% for adipose tissue estimated by the BIS method), compared with assessments at the individual level (95% limits of agreement, LOA, of −8.52 to 8.95% for adipose tissue estimated by the BIS method). The BIS method is therefore particularly well‐suited for field‐based population assessments. Despite the larger LOA for individual measurements (i.e., lower accuracy at the individual level), since intra‐animal variability was extremely low and precision of measurement very high (Kophamel et al., 2023), BIS is suitable to assess changes in adipose tissue in individual turtles over time, such as repeated sampling of adult females during nesting season and turtles temporarily held in rehabilitation centres or permanently living in captivity (e.g., aquaria). While the mean difference between methods (bias) represents accuracy at the population level, the limits of agreement indicate confidence in method agreement for an individual (Figure 4; Table 1; Appendix S1, Figure A2). Since the precision of the technique is extremely high, BIA can be used to assess adipose tissue data in individual turtles over time even if individual accuracy (agreement with the reference method) is relatively weak. As an example, if a turtle has 2% of adipose tissue and this value increases to 3% in the next season, the BIS device will still pick up on the 1% increase even if the estimated absolute adipose tissue does not match the true absolute value. In other words, BIA can detect a 1% change in adipose tissue, even if the agreement to adipose tissue estimated by CT scans is lower for individuals, compared with the population level. Animal health professionals could thus use the BIS technique with confidence to determine whether the turtles are gaining or losing adipose tissue over time. The implications of tracking adipose tissue over time are manyfold: in nesting females, the link between adipose tissue and reproductive success could be further explored by using BIA; in rehabilitating turtles, adipose tissue might provide details on treatment efficacy and/or dietary supplementation; and in captive animals, which commonly suffer from obesity and resulting liver problems (Stacy & Innis, 2017; Stewart et al., 2016), adipose tissue levels could be compared to those of wild animals to adjust their diet accordingly.

The LOA found here are similar to those previously reported in human clinical studies using diagnostic imaging as a calibration method (Forde et al., 2015; Tewari et al., 2018; Zopfs et al., 2020). Agreement metrics (mean bias and LOA) varied depending on impedance parameters; with Rinf, R50, and R0 providing the closest agreement. We suggest using the impedance index length^2^/R50, since this can be obtained using the simpler and less expensive single‐frequency (i.e., 50 kHz) bioelectrical impedance devices. Consequently, Equation 2 is recommended for use in future studies to quantify body composition from impedance measurements. BIS devices, in contrast to single‐frequency devices, use a range of frequencies to measure impedance and allow the quantification of body water compartment volumes, which is of importance in human clinical studies (Kyle et al., 2004; Yamada et al., 2013). For adipose tissue estimation based on total body water, however, single‐frequency devices are considered as suitable as BIS devices (Brantlov et al., 2017).

Calibration of the BIS device was conducted by using deep learning, which enabled the automated adipose tissue identification and quantification. Deep learning approaches in ecology facilitate the classification, regression, and modeling of data (Borowiec et al., 2022). For example, deep learning has been used to identify, classify, and estimate the density of individuals, populations, and species (Christin et al., 2019). The resulting predictive models have enabled the conducting of diversity assessments and have supported conservation and resource management projects (Christin et al., 2019). Deep learning approaches that were originally developed for humans can be easily transferred to other species, since the modeling aspect would not significantly change (Ditria et al., 2020). In some cases, the performance levels of vertebrate wildlife species have outperformed those of human studies (Traore et al., 2018). In comparison to conventional machine learning processes, deep learning enables an automated feature extraction from large amounts of input data (e.g., CT scans), which overcomes the time limitations of semi‐automated and manual approaches. The multifactorial applicability of deep learning in ecology and the high performance in identification and classification tasks make this technique highly attractive for developing and/or validating new methods for threatened species research.

BIS was over 50 times more accurate in predicting adipose tissue at the population level, compared with solely using body mass, from which BCI is derived. Moreover, the precision of BIS was consistent with repeatability values reported in human clinical studies (Kophamel et al., 2023). The high precision and accuracy of BIS in green turtles confirm the suitability of the technique for sea turtle monitoring programs and endorse BIS as a robust alternative to conventional morphometric measures and BCI.

### 4.1 Future directions and study limitations

*Adipose tissue prediction at the individual level—*A disadvantage of the BIS method is that the accuracy of adipose tissue prediction at the individual level was significantly lower than at the population level. Adipose tissue cannot be reliably determined in individual turtles when the determination error is larger than the typical absolute adipose tissue value. Conversely, the high accuracy of impedance measurements at the population level, in comparison to solely using body mass as predictor variable for adipose tissue, highlights its suitability to estimate adipose tissue across sea turtle populations or to assess differences across foraging or nesting aggregations. However, since intra‐animal variability was extremely low and thus the precision of measurement was very high (Kophamel et al., 2023), BIS can be used to  assess changes in adipose tissue in individual turtles over time (i.e., repeated sampling).

*Animal selection—*Our study was limited to animals with a maximum carapace width of 55 cm due to the gantry size of the CT scanner (typical CT scanner found in human imaging centres; limited access to CT scanners with larger gantry sizes and close to the turtle capture sites), and due to the inability of obtaining body composition data in a nondestructive manner by using another method. We assumed that the parametrization of the model would not significantly change with the inclusion of larger turtles and that any extrapolation of the procedure to full‐size adult turtles would be predicated on the assumptions of proportionality of body change. Although the proposed methods can be transferred to larger animals and to other species, future studies might benefit from testing the calibration protocol across a wide range of life stages that represent the broad range of body sizes seen in the population.

*Sample size and cross‐validation*—Funding, permitting, and time constraints restricted sample size and cross‐validation. The sample size used in our study was limited (*n =* 49 animals), and cross‐validation should ideally be undertaken in a completely independent population. Random data splitting was used instead of cross‐validation (Stevens, 2013). Nevertheless, the small mean biases and acceptable LOA confirmed the accuracy of the calibration for impedance indices length^2^/R0, length^2^/R50, length^2^/Rinf, and length^2^/Ri (Smith Jr. et al., 2009). The maximum allowed difference indicated that our sample size would allow detection of a difference in adipose tissue mass of <0.8 kg for impedance indices length^2^/R0, length^2^/R50, length^2^/Rinf, and length^2^/Ri (Lu et al., 2016). We also confirm that the accuracy of the technique was especially high for population assessments (mean bias of 0.11% to 0.61% for impedance indices).

*Calibration against a single reference method—*We calibrated the BIS device against a single reference method (i.e., CT scans) due to species constraints, funding, permitting, and time limitations. CT scanning was chosen as a calibration method since it shows a similar accuracy to chemical analyses and dilution methods for body composition assessment (Ishioka et al., 2005; Kim et al., 2018; Kobayashi et al., 2013). In addition, CT scans are noninvasive, which is highly advantageous when working with threatened species. Other in vivo methods proposed for adipose tissue estimation in vertebrates are dilution methods using tracers (Nagy, 1989; Pagano & Williams, 2019; Shaffer, 2011). The suitability of each method depends on the target species and should be determined with caution. Reptiles in particular pose additional challenges, as dilution methods, such as doubly‐labeled water methods, are confounded by high water flux in some species (e.g., sea turtles; Jones et al., 2009; Price, 2017). Additional ethical or regulatory (access in protected species) constraints such as the degree of invasiveness, the need for anesthesia, and sampling duration might further hinder the collection of biological data. Diagnostic imaging tools such as CT scans or magnetic resonance imaging are increasingly used as alternative methods to estimate the body composition of wildlife and companion animals (Barba et al., 2018; Clelland et al., 2018; De Persio et al., 2019; Eastick et al., 2021; Gimmel et al., 2020; Kim et al., 2018). CT is widely established in human and veterinary medicine and provides reliable results for adipose tissue mass estimation (Mattsson & Thomas, 2006; Zopfs et al., 2020). Due to these reasons, we decided to use CT scans as the calibration method of the BIS device.

*Accuracy of the CNN architecture—*The trained U‐Net semantic‐segmentation CNN architecture is only as accurate as the masks they were trained on. The creation of highly accurate training masks is a very time‐consuming process (i.e., an operator might need 2–3 weeks of full‐time work per animal). Segmentation of CT images was therefore trained on only three animals to ensure the highest possible training quality.

*Misclassified tissue areas—*Due to similar tissue densities, areas of the gastrointestinal tract might have been misclassified as adipose tissue, and adipose tissue areas of the mesenteric region might have been missed. This is a clear limitation of the CNN approach. To address this issue, all adipose tissue masks resulting from the CNN procedure were visually confirmed for each animal. Masks that did not correctly display the adipose tissue areas were edited, and the algorithm was re‐fit until the visual assessment of the final masks was satisfactory. Therefore, the number of misclassified pixels can be assumed negligible.

## 5 CONCLUSIONS

The proposed BIS method represents an improvement over current methods due to its quantitative nature, higher accuracy, and tissue‐specificity for body composition assessment in sea turtles and potentially other species. Bioelectrical impedance devices are extremely time‐efficient once the final prediction equations for the target species have been established and are relatively affordable ($1–10 k AUD for a portable device). Our approach will help to identify changes in nutritional status across populations and can support timely and effective conservation action in sea turtles and other vertebrate wildlife.

## AUTHOR CONTRIBUTIONS

**Sara Kophamel:** Conceptualization (lead); data curation (lead); formal analysis (lead); funding acquisition (lead); investigation (lead); methodology (lead); project administration (lead); resources (lead); software (lead); validation (lead); visualization (lead); writing – original draft (lead); writing – review and editing (lead). **Leigh C. Ward:** Conceptualization (supporting); formal analysis (supporting); methodology (supporting); supervision (equal); validation (supporting); writing – review and editing (supporting). **Dmitry A. Konovalov:** Conceptualization (supporting); formal analysis (supporting); methodology (supporting); software (supporting); validation (supporting). **Diana Mendez:** Conceptualization (supporting); writing – review and editing (supporting). **Ellen Ariel:** Conceptualization (supporting); data curation (supporting); methodology (supporting); project administration (supporting). **Nathan Cassidy:** Data curation (supporting); methodology (supporting); resources (supporting). **Ian Bell:** Data curation (supporting); resources (supporting). **María T. Balastegui Martínez:** Methodology (supporting); validation (supporting). **Suzanne L. Munns:** Conceptualization (supporting); methodology (supporting); project administration (supporting); supervision (equal); validation (supporting); writing – review and editing (supporting).

## FUNDING INFORMATION

Funding for SK was provided by James Cook University [International Postgraduate Research Scholarship] and Sea World Research and Rescue Foundation [SWR/6/2019].

## CONFLICT OF INTEREST

The funders of the study had no role in study design, data collection and analysis, decision to publish, or preparation of the manuscript. The authors declare no conflict of interest.

### OPEN RESEARCH BADGES

This article has earned an Open Data badge for making publicly available the digitally‐shareable data necessary to reproduce the reported results. The data is available at https://doi.org/10.25903/gzf1‐8e56.

## Supporting information


Appendix S1

Figure A1

Figure A2

Figure A3

## Data Availability

The datasets for assessing the validity of our work (.xlsx, .ods., and .csv formats) are available at James Cook University Data Repository under the following link: https://doi.org/10.25903/gzf1‐8e56 [doi:10.25903/gzf1‐8e56] (Kophamel, Ward, et al., 2022).
