# Supplementary material for: Field‐based adipose tissue quantification in sea turtles using bioelectrical impedance spectroscopy validated with CT scans and deep learning
Source: Ecol Evol. 2022 Dec 13;12(12):e9610. doi: 10.1002/ece3.9610 (PMC9748411; doi:10.1002/ece3.9610)
Supplement: Supplementary file 1 — Appendix S1 [file ECE3-12-e9610-s003.pdf]

Appendix from: Field-based adipose tissue quantification in sea turtles using bioelectrical impedance spectroscopy validated with CT scans and deep learning

Table A1. List of abbreviations used in the manuscript, sorted by categories.

Abbreviation	Definition
Bioelectrical Impedance Spectroscopy	
BIS	Bioelectrical Impedance Spectroscopy
FM	Fat-mass
FFM	Fat-free mass
AT	Adipose tissue (percentage)
Rinf	Resistance at infinite frequency (ohm)
R50	Resistance at 50 kHz (ohm)
R0	Resistance at zero frequency (ohm)
Ri	Intracellular resistance (ohm)
Xc50	Reactance at 50 kHz (ohm)
PhA50	Phase angle at 50 kHz (ohm)
Animal characteristics	
SCL	Straight carapace length (cm)
CCL	Curved carapace length (cm)
Automated segmentation of computed tomography images	
CT	Computed tomography
kVp	Peak kilovoltage
mA	Milliamperage
CNN	Convolutional neural networks
HU	Hounsfield units
DICOM	Digital Imaging and Communications in Medicine
VGG	Visual geometry group
GIT	Gastrointestinal tract
Statistical analyses	
GAM	Generalised additive models
AICc	Corrected Akaike Information Criterion
MAPE	Mean absolute percentage error

Table A2. Characteristics of the examined green turtles (*Chelonia mydas*) (mean ± standard deviation). (CCL) curved carapace length (cm); (SCL) straight carapace length (cm); (BCI) body condition index*.

Parameter	Values
Sample size	49
CCL (mean cm ± SD)	37.9 ± 10.8
SCL (mean cm ± SD)	36.6 ± 10.8
Mass (mean kg ± SD)	7.1 ± 5.3
BCI	1.22 ± 0.15
Cloacal temperature (mean ^{o}C ± SD)	28.5 ± 2.4
Ambient temperature (mean ^{o}C ± SD)	26.2 ± 2.6

* BCI calculation sourced from Bjorndal et al. (2000), i.e., Fulton's condition factor K = body mass/length^3 * 10,000.

Figure legends of the figures provided as supplementary files

Figure A1. Visual summary of the identification of adipose tissue Hounsfield units (HU) in a green turtle (*Chelonia mydas*); transverse and dorsal planes (NovaPACS, Novarad™, American Fork, US). Threshold-based technique to isolate (a) areas with a lower attenuation than adipose tissue; (b) areas with a higher attenuation than adipose tissue; (c) total body region, identified by summing (a) and (b); (d) non-corrected adipose tissue, identified by subtracting (b) from (c); (e) corrected adipose tissue after manual exclusion of the gastrointestinal contents. The resulting adipose tissue HU were extracted from the final mask (e).

Figure A2. Bland and Altman plots of the differences in adipose tissue estimations using computed tomography and bioelectrical impedance spectroscopy (BIS) in green turtles (*Chelonia mydas*). (a-h) Predictor variables were resistance (R) index calculated using resistance at infinite frequency (Rinf), resistance at zero frequency (R0), resistance at 50 kHz (R50), and intracellular resistance (Ri), as well as straight carapace length (SCL; a, c, e, g) or curved carapace length (CCL; b, d, f, h). To assess the accuracy of body mass as sole predictor variable for adipose tissue mass estimation, (i) an additional model using body mass as predictor variable (excluding impedance index) was created for comparison purposes with the best fitting model (e and f). The Bland and Altman plot quantifies the bias and range (i.e., limits) of agreement that includes 95% of the differences between two methods (Altman and Bland 1983, Bland and Altman 1986). While the mean difference between methods (bias) represents accuracy at the population level, the limits of agreement indicate confidence in method agreement for an individual. The regression line (blue solid line) and its 95% confidence interval (grey area) show the relationship between the dependent variables on the Y axis (differences between AT estimates from CT and BIS measurements) and the independent variables on the X axis (mean of each pair of AT estimates from CT and BIS measurements). (e) The lowest mean bias (0.11%) and 95% limits of agreement (-8.52% lower and 8.95% upper) were generated using straight carapace length^2/resistance at 50 kHz ($SCL^2/R50$) as predictor variable (Figure 4 and Table 1). Please refer to Giavarina (2015) for a detailed explanation on how to use and interpret Bland and Altman plots.

Figure A3. Passing and Bablok regression plots of the analytical methods agreement between computed tomography (Method 1) and bioelectrical impedance spectroscopy (Method 2) for adipose tissue estimation in green turtles (*Chelonia mydas*). (a-h) Predictor variables were resistance (R) index calculated using resistance at infinite frequency (Rinf), resistance at zero frequency (R0), resistance at 50 kHz (R50), and intracellular resistance (Ri), as well as straight carapace length (SCL) or curved carapace length (CCL). (i) Body mass used as sole predictor variable for adipose tissue estimation. The 0.95-confidence bounds were calculated with the bootstrap (quantile) method. These plots show the distribution of the samples (dots) and the measurement errors (blue line) (Passing and Bablok 1983). The slope and the intercept were calculated with 95% confidence levels.

Description of parameters examined and codes used in the dataset

The datasets for assessing the validity of our work (.xlsx, .ods., and .csv formats) are available at James Cook University Data Repository under the following link: https://doi.org/10.25903/gzf1-8e56 [doi:10.25903/gzf1-8e56] (Kophamel et al. 2022b).

Parameter	Unit	Description
id		Animal ID
date		Date of sampling (year/month/day)
location		Location (Captive animals ["caraplace"]; Cockle Bay, and Toolakea Beach)
ccl	centimetres	Curved carapace length (CCL)
scl	centimetres	Straight carapace length (SCL)
weight	kilogram	Total body mass
bci		
temp_air	celsius	Air temperature
temp_cloaca	celsius	Cloacal temperature
postfeeding_h	hours	Hours after feeding
bia_time		Time at which the bioelectrical impedance spectroscopy (BIS) measurements were performed (hours:minutes)
bia_postcapt_h	hours	Hours passed between capture time and BIS examination
R0	ohm	Resistance at zero frequency
R0_sd	ohm	Standard Deviation SD of Resistance at zero frequency (R0)
R0_scl_meas		Impedance index, calculated using R0 and SCL
R0_ccl_meas		Impedance index, calculated using R0 and CCL
Rinf	ohm	Resistance at infinite frequency
Rinf_sd	ohm	Standard Deviation SD of Resistance at infinite frequency (Rinf)
Rinf_scl_meas		Impedance index, calculated using Rinf and SCL
Rinf_ccl_meas		Impedance index, calculated using Rinf and CCL
ph50	degrees	Phase angle at 50 kHz
ph50_sd	degrees	Standard Deviation SD of Phase angle at 50 kHz (ph50)
R50	ohm	Resistance at 50 kHz
R50_sd	ohm	Standard Deviation SD of Resistance at 50 kHz (R50)
R50_scl_meas		Impedance index, calculated using R50 and SCL
R50_ccl_meas		Impedance index, calculated using R50 and CCL
Ri	ohm	Intracellular Resistance
Ri_sd	ohm	Standard Deviation SD of Intracellular Resistance (Ri)
Ri_scl_meas		Impedance index, calculated using Ri and SCL
Ri_ccl_meas		Impedance index, calculated using Ri and CCL
Xc50	ohm	Reactance at 50 kHz
Xc50_sd	ohm	Standard Deviation SD of Reactance at 50 kHz (Xc50)
Xc50_scl_meas		Impedance index, calculated using Xc50 and SCL
Xc50_ccl_meas		Impedance index, calculated using Xc50 and CCL
AI_body_countVolume	cm^3	Total body volume in cubic centimetres
AI_fat_countVolume_HUsInd	cm^3	Total fat volume in cubic centimetres
AI_fat_perc_HUsInd	percentage	Total adipose tissue in percentage
AI_fat_mass_HUsInd	kilogram	Total adipose tissue in kilogram
AI_nonadipose_mass_HUsInd	kilogram	Total non-adipose tissue in percentage
AI_nonadipose_perc_HUsInd	percentage	Total non-adipose tissue in kilogram
