# Supplementary figures and images for: Field‐based adipose tissue quantification in sea turtles using bioelectrical impedance spectroscopy validated with CT scans and deep learning
Source: Ecol Evol. 2022 Dec 13;12(12):e9610. doi: 10.1002/ece3.9610 (PMC9748411; doi:10.1002/ece3.9610)

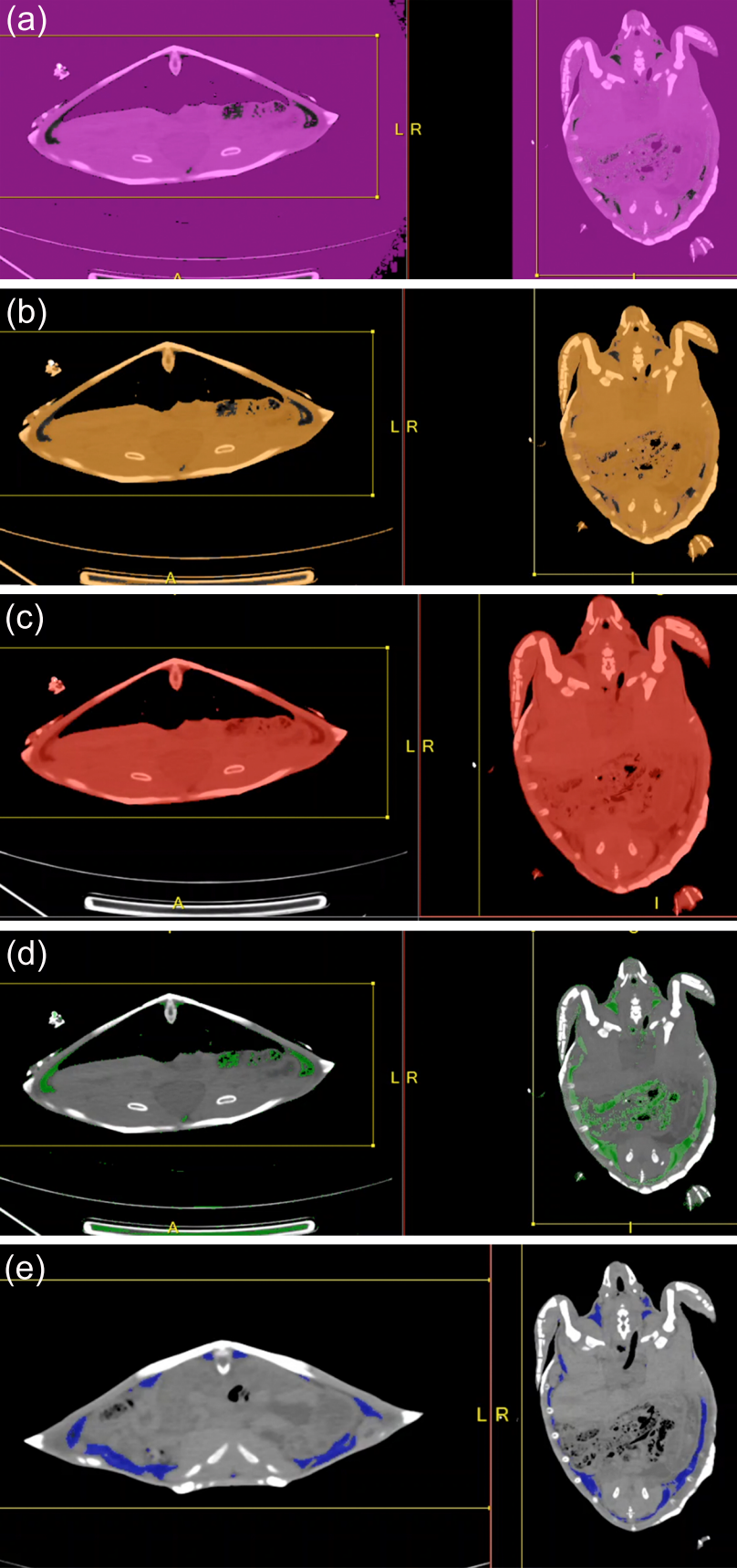

Supplement: Supplementary file 2 — Figure A1 [file ECE3-12-e9610-s004.pdf]

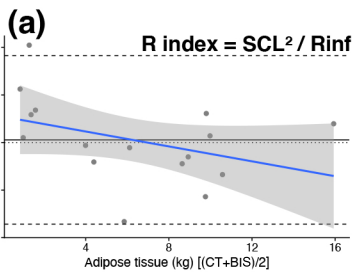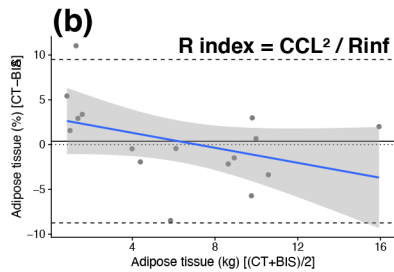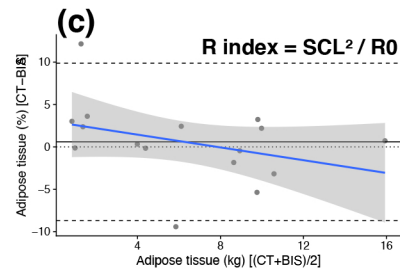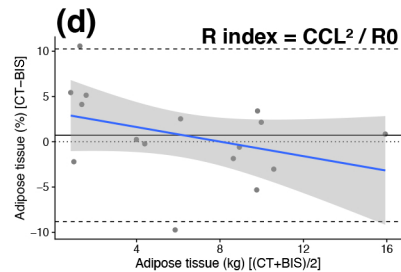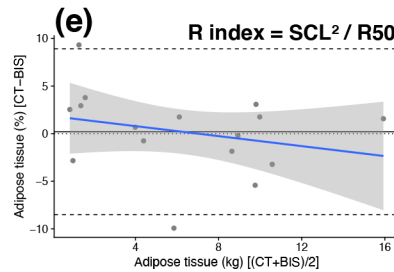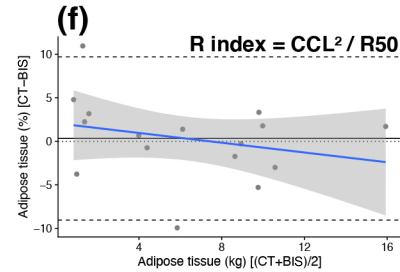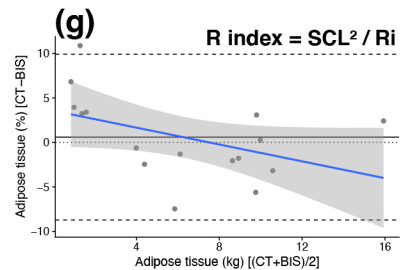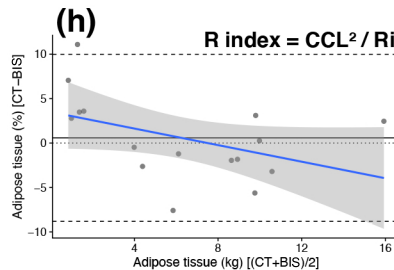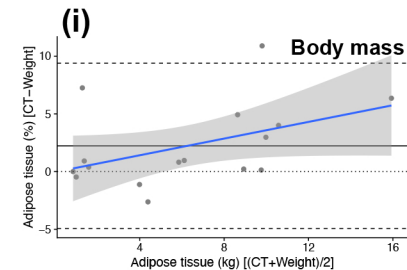

Supplement: Supplementary file 3 — Figure A2 [file ECE3-12-e9610-s001.pdf]
