# Supplementary material for: Field‐based adipose tissue quantification in sea turtles using bioelectrical impedance spectroscopy validated with CT scans and deep learning
Source: Ecol Evol. 2022 Dec 13;12(12):e9610. doi: 10.1002/ece3.9610 (PMC9748411; doi:10.1002/ece3.9610)
Supplement: Supplementary file 4 — Figure A3 [file ECE3-12-e9610-s002.pdf]

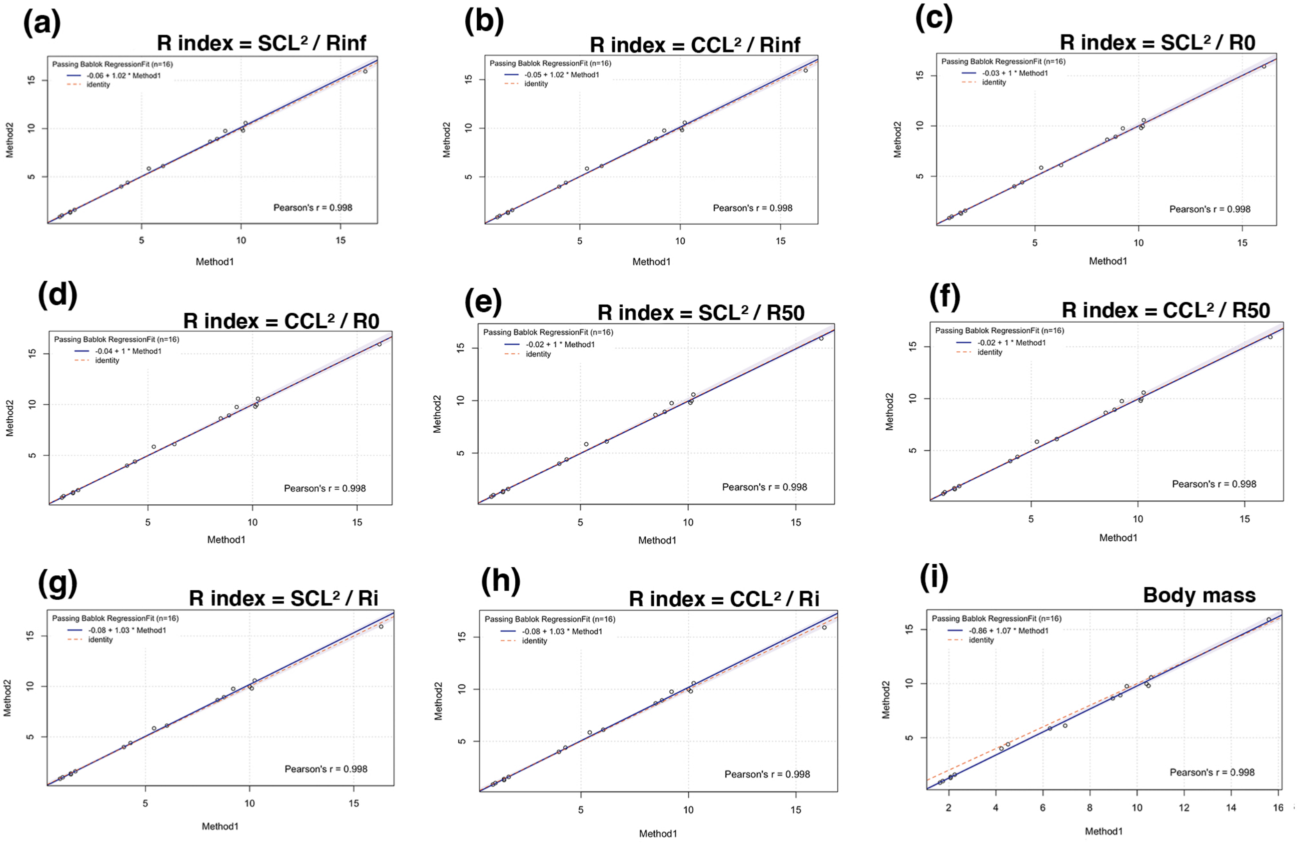
(a)
R index = SCL² / Rinf
Passing Bablok RegressionFit (n=16)
-0.06 + 1.02 * Method1
identity
Pearson's r = 0.998
(b)
R index = CCL² / Rinf
Passing Bablok RegressionFit (n=16)
-0.05 + 1.02 * Method1
identity
Pearson's r = 0.998
(c)
R index = SCL² / R0
Passing Bablok RegressionFit (n=16)
-0.03 + 1 * Method1
identity
Pearson's r = 0.998
(d)
R index = CCL² / R0
Passing Bablok RegressionFit (n=16)
-0.04 + 1 * Method1
identity
Pearson's r = 0.998
(e)
R index = SCL² / R50
Passing Bablok RegressionFit (n=16)
-0.02 + 1 * Method1
identity
Pearson's r = 0.998
(f)
R index = CCL² / R50
Passing Bablok RegressionFit (n=16)
-0.02 + 1 * Method1
identity
Pearson's r = 0.998
(g)
R index = SCL² / Ri
Passing Bablok RegressionFit (n=16)
-0.08 + 1.03 * Method1
identity
Pearson's r = 0.998
(h)
R index = CCL² / Ri
Passing Bablok RegressionFit (n=16)
-0.08 + 1.03 * Method1
identity
Pearson's r = 0.998
(i)
Body mass
Passing Bablok RegressionFit (n=16)
-0.86 + 1.07 * Method1
identity
Pearson's r = 0.998
Method1
Method2
